# ncRNAs in Type-2 Immunity

**DOI:** 10.3390/ncrna6010010

**Published:** 2020-03-06

**Authors:** Riccardo Guidi, Christopher J. Wedeles, Mark S. Wilson

**Affiliations:** Genentech Inc, South San Francisco, CA 94080, USA; guidir1@gene.com (R.G.); wedelesc@gene.com (C.J.W.)

**Keywords:** miRNA, LncRNA, helminth, allergy, IL-4, IL-13, T_H_2

## Abstract

Immunological diseases, including asthma, autoimmunity and immunodeficiencies, affect a growing percentage of the population with significant unmet medical needs. As we slowly untangle and better appreciate these complex genetic and environment-influenced diseases, new therapeutically targetable pathways are emerging. Non-coding RNA species, which regulate epigenetic, transcriptional and translational responses are critical regulators of immune cell development, differentiation and effector function, and may represent one such new class of therapeutic targets. In this review we focus on type-2 immune responses, orchestrated by T_H_2 cell-derived cytokines, IL-4, IL-5 and IL-13, which stimulate a variety of immune and tissue responses- commonly referred to as type-2 immunity. Evolved to protect us from parasitic helminths, type-2 immune responses are observed in individuals with allergic diseases, including Asthma, atopic dermatitis and food allergy. A growing number of studies have identified the involvement of various RNA species, including microRNAs (miRNA) and long non-coding (lncRNA), in type-2 immune responses and in both clinical and pre-clinical disease settings. We highlight these recent findings, identify gaps in our understanding and provide a perspective on how our current understanding can be harnessed for novel treat opportunities to treat type-2 immune-mediated diseases.

## 1. Introduction

### 1.1. Innate and Adaptive Collaboration

**Figure 1:**
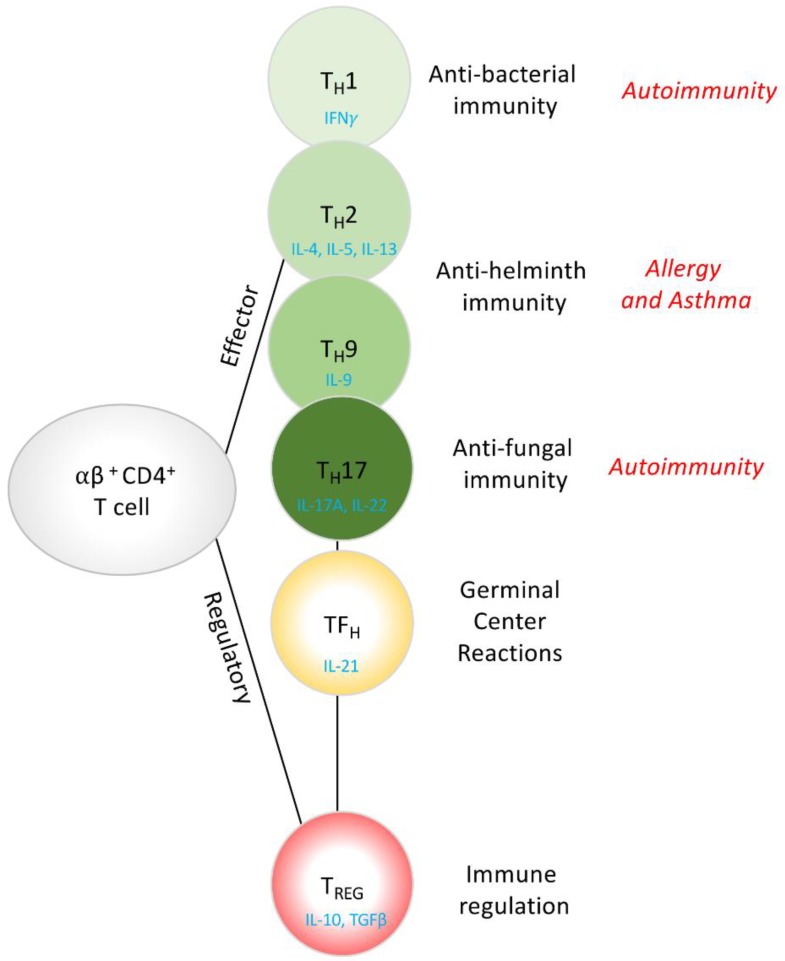
Naïve CD4^+^ T cells differentiate, in the thymus or periphery, into a variety of effector or regulatory phenotypes. The current model of T cell differentiation can be appreciated through their function, with IFNγ-secreting T_H_1 cells providing protection from intracellular pathogens, including bacteria, viruses, and parasitic protozoa. IL-4, IL-5 and IL-13-secreting T_H_2 cells, and IL-9-secreting T_H_9 cells providing protection from extracellular pathogens including parasitic helminths, IL-17A and IL-22-secreting T_H_17 cells providing protection from extracellular pathogens including fungal infections. IL-21-secreting TF_H_ cells help orchestrate the germinal center for B cell activation and antibody production and finally, IL-10 and TGFβ-secreting T_REG_ cells providing regulation of adaptive and innate immune responses via suppressive mechanisms. Dysregulated T cell responses can give rise to Autoimmunity, Allergy and Asthma.

Highly evolved and constantly adapting to the changing environment, the immune system is tightly regulated in mammals, protecting us from a barrage of both infectious and non-infectious insults. The activation of an immune response can be loosely broken down into a bi-phasic innate and adaptive ‘reply’ to an insult. Following tissue damage, tissue-derived ‘alarmins’, including thymic Stromal Lymphopoietin (TSLP), IL-33 and IL-25, or danger signals activate innate immune cells, which respond by secreting detoxifying molecules in an attempt to kill, neutralize and prevent the spread of an infectious agent. The innate reaction is also responsible for initiating the cardinal features of inflammation-*rubor* (redness), *calor* (increased heat), *tumor* (swelling), *dolor* (pain) and *laesa* (loss of function). In addition to alarmins, recognition of *pathogen associated molecular patterns* (PAMPs) by *pathogen recognition receptors* (PRR) activates innate immune cells, which relay pathogen-specific information to the adaptive immune system. Pathogen-specific information, including the secretion of pathogen-relevant cytokines and the presentation of fragments of the invading pathogen to a pool of pre-existing pathogen-specific CD4^+^ T cells, stimulates the activation, expansion and differentiation of T cells into effector T cells (Figure 1). Important feed-forward functions of the adaptive T cell response mobilize a second wave of innate cells, provide help to B cells for immunoglobulin (Ig) class switching and antigen-specific Ig production, provide cues to local tissue, and promote wound healing and tissue repair. With such broad functions, CD4^+^ T cells need to be tightly regulated throughout their development, differentiation, expansion and ultimately their effector function. Despite multiple checkpoints and layers of self-governing immune regulation, CD4^+^Th cell dysfunction can arise, leading to hyper-inflammatory conditions in response to self-antigens (autoimmunity) or exogenous innocuous antigens (such as allergic diseases). Conversely, if CD4^+^Th cells fail to develop, mature, activate or differentiate, individuals can be left with insufficient immunological protection with equally catastrophic outcomes, such as life-threatening severe immunodeficiency. 

### 1.2. CD4^+^ T Cells, Conductors of the Immunology Orchestra

The immune system has evolved to mount an appropriate and distinct innate and adaptive response to different classes of pathogens. The differentiation of CD4^+^ T_H_ cells from naïve into effector or regulatory T cells requires the ligation of the T cell receptor (TCR) by antigen bound MHC molecules on innate antigen-presenting cells (APC), with appropriate co-stimulation and cytokine receptor engagement. CD4^+^ T_H_ cells differentiate into at least five, if not six, CD4^+^T cell subsets including four effector T cell populations (T_H_1, T_H_2, T_H_9, T_H_17) [1,2], follicular helper T cells (T_FH_) and regulatory T cells (T_REG_), characterized by their cytokine expression profile, transcription factor usage and most importantly, their function. It is important to note that plasticity between the subsets is also now widely documented and accepted with many studies identifying T_H_2 (GATA3^+^IL-4^+^) cells that either co-express or fully convert to express T_H_1-defining features (T-bet and IFNγ) [2], T_H_2 cells that convert to express T_H_17-defining features (RoRγt and IL-17A) [3], T_H_2 cells that up-regulate markers of T_H_9 (IL-9-secretion) [4] or T_H_2 cells that convert to express T_REG_-defining features, including Foxp3 [5,6], to name a few.

When viewed through functional optics, the different effector T cell populations provide appropriate protection from a variety of pathogens; IFNγ-producing T_H_1 cells (which also produce TNFα, granzymes, perforins and a suite of chemokines) potently activate pathways involved in the killing of intracellular pathogens including parasitic protozoa, bacteria and viruses. IL-4-producing T_H_2 cells (which also secrete IL-5 and IL-13 and a different suite of chemokines) activate local stroma, mobilize and activate innate immune cells and are required for killing extracellular pathogens including large multi-cellular parasitic worms [7,8]. IL-17A-producing T cells appear to have evolved to control extracellular fungal infections, limiting their growth and spread [9,10] while the precise function of T_H_9 cells is still evolving, their contribution to type-2 allergic like responses is emerging [11]. Follicular helper T cells (T_FH_) emerged as a distinct T cell population required for assembly of the germinal center (GC) reaction for appropriate B cell help and antibody production [12]. Regulatory T cells (T_REG_), which are a distinct T cell lineage that can develop in the thymus (tT_REG_ cells) or periphery (pT_REG_ cells), oppose many of the actions of the effector T cell subsets and provide a T cell intrinsic regulatory arm, counterbalancing the activation and expansion of effector T cell responses [13].

### 1.3. CD4^+^ T_H_2 Cell Differentiation

For T_H_2 cell differentiation, IL-4 receptor ligation provides the 3rd necessary extracellular cue, in addition to TCR engagement and co-stimulation, to initiate a transcriptional re-organization required for T_H_2 cell identity. Both dendritic cells and basophils have emerged as important IL-4-secreting and antigen-presenting innate cells required for optimal T_H_2 cell differentiation [14,15,16,17,18,19,20,21,22,23]. The importance of co-stimulatory molecules on T cells was realized when antigen presented on MHC II molecules alone was insufficient to fully stimulate CD4^+^T cells. It was later discovered that costimulatory molecules (CD80 and CD86) on APCs interact with CD28 on T cells, initiating distinct and necessary signaling events for optimal T cell activation. In addition to CD28, the CD28 superfamily includes a suite of activation and inhibitory receptors, including ICOS, CTLA-4 and PD-1, providing an important level of regulation, governing T cell activation, and regulating T_H_2 cell differentiation [24,25,26,27,28].

The intracellular molecular events required for T_H_2 cell differentiation are relatively well understood. IL-4Rα-driven signaling is transduced by the transcription factor STAT-6, which in combination with TCR-derived signals and other transcription factors, activates NFAT and GATA-binding protein-3 (GATA-3) [29]. *Il4*, *Il5*, *Il13,* within the *Il4* locus are transcribed with IL-2 produced as a result of TCR triggering leading to STAT5-dependent IL-4 production [30]. GATA3 is both necessary and sufficient for T_H_2 development and lies at the heart of T_H_2 cell differentiation and proliferation. Transgenic over-expression of *gata3* induces *il4* [31] while its absence abolishes *il4* transcription [7,32]. GATA3 also serves to stabilize chromatin rearrangement within the *il4* locus during T_H_2 differentiation [33,34].

In addition to IL-4, the ‘alarmins’, produced by a variety of cells, including epithelial cells [35,36,37], mast cells [38], antigen presenting cells (APC) [39] and basophils [15,40,41], can act on both innate cells, including innate lymphoid cells (ILC) and APCs and T cells [42,43,44,45,46,47,48,49,50], participating directly and in-directly in T_H_2 cell differentiation [51,52]. Furthermore, TSLP, IL-25 and IL-33 can re-enforce T_H_2 responses, either by recruiting more IL-4-producing basophils [17] or other T_H_2 cells [53] or by up-regulating GATA3 in T cells [54]. Thus, IL-4, TSLP, IL-25 and IL-33 have all been associated with differentiation, activation and/or recruitment of T_H_2 cells.

Following T_H_2 differentiation, chromatin remodeling at conserved non-coding sequence-1 (CNS-1), DNase I hypersensitivity (DHS) site, CNS-2, and the conserved intron 1 sequence of IL-4 (CIRE) in the *il4* locus facilitates rapid cytokine transcription [55,56,57]. Poised in such a state, it may only require a low level of T cell activation to induce translation and secretion of type-2 cytokines.

### 1.4. T_H_2 Cell Effector Function and Type-2 Immunity

T_H_2 cell-derived cytokines stimulate a variety of responses- commonly referred to as type-2 immunity. IL-4 can drive IgG1, IgG4 and IgE class switching in B cells [58,59], alternative-activation of macrophages [60] and stabilize T_H_2 cells; IL-5 mobilizes, matures [61] and recruits innate effector cells, such as eosinophils [62] and IL-13 induces goblet cell differentiation, mucus secretion and local tissue repair and/or fibrosis [63]. Although T_H_2 cells provide this trio of type-2 cytokines, ILC2 cells also secrete these cytokines. Functionally, these cytokine-driven events initiate the necessary cellular and tissue responses required to expel intestinal helminths. Similarly, this inflammatory response is seen in many patients with atopic asthma, characterized by the release of IL-4, IL-5, IL-13 [64], the induction of mucus-hypersecretion and the remodeling of the lung- very similar to the anti-helminth response. These observations have focused researchers on developing strategies to prevent T_H_2 cell development and target type-2 cytokines and effector functions to prevent allergen-driven pathologies. Although therapeutic approaches are being developed to target type-2 immune responses (reviewed in [65]), there are still significant gaps in our knowledge. Regulating the events leading to T_H_2 cell differentiation, T_H_2 cell survival and migration, T_H_2 cell effector function and cytokine secretion occurs at many levels. This review will present our current understanding of the role of long non-coding RNAs (lncRNAs) and microRNAs (miRNAs) in regulating type-2 immunity and provide a prospective on how we may be able to manipulate type-2 immunity by targeting these non-protein coding RNA species.

## 2. Long Non-Coding RNAs (LncRNA) in Type-2 Immunity

Long non-coding RNAs (lncRNAs) are a diverse class of non-coding RNAs defined simply as a non-coding transcript greater than 200 nucleotides in length [66]. lncRNAs can be categorized into distinct subsets based on their position relative to adjacent protein coding genes: long intergenic noncoding RNAs, intronic lncRNAs, antisense lncRNAs and enhancer RNA (eRNAs) [66,67]. As this categorization suggests, the genomic positions of lncRNA coding sites ranges from within introns to over 50 kb from the nearest protein-coding transcript. The vast majority of lncRNAs are transcribed and processed in a manner that is analogous to canonical protein-coding mRNAs [68]. lncRNA loci resemble protein coding gene loci and may possess conserved promoters, characteristic histone modifications and transcription factor binding sites [69]. A significant fraction of lncRNAs are transcribed in an RNA polymerase II (RNAPII)-dependent process that involves capping, splicing and poly-adenylation. Despite these general similarities, lncRNA promoters are enriched for H3K9me3 relative to the promoters of expression matched mRNAs and contain fewer exons that are inefficiently spliced [70].

As a class, lncRNA encoding sequences are poorly conserved relative to protein-coding genes. In 2014 Necsulea et al. performed a large-scale evolutionary study of lncRNA in tetrapods ranging from frogs to primates and humans [71]. While a number of highly conserved lncRNAs do exist, this study (and others like it) revealed poor sequence conservation of lncRNAs [72]. Of over 13,000 lncRNAs considered, 81% were primate-specific. To this end, 92% of human intergenic lncRNAs are also expressed in chimpanzees or bonobo (72% were expressed in macaques) which contrasts an overlap of 98% when considering protein coding genes. Importantly, this analysis also revealed the presence of hundreds of ancient lncRNAs, that arose at least 300 million years ago. Of critical importance is the observation that lack of sequence conservation does not equate to lack of function, as even poorly conserved lncRNA have been shown to be functional [73]. It may be that the structure of the lncRNAs are under evolutionary constraint, or that short fragments within the transcripts are highly conserved whereas the remainder of the transcript is free to diverge, or it is the act of the lncRNA being transcribed that is under selective pressure versus the sequence itself (this will be discussed in more detail below).

Since their discovery approximately a decade ago, dysregulated lncRNAs have been identified in numerous human pathologies, including cancer, autoimmunity and asthma. For example, RNA isolated from whole blood of individuals with eosinophilic asthma revealed that 27 lncRNAs were at-least 2-fold dysregulated in diseased individuals relative to controls [74]. While the sample size was small (six asthma patients and three healthy individuals), re-analysis of larger cohorts of patients would add confidence to these findings. Similarly, identifying the cell types responsible for the mis-regulation of individual lncRNAs would be necessary to begin understanding their contribution to disease. Because of the limited number of studies directly related to lncRNA in type-2 inflammation, the remainder of this section will focus on broad concepts central to lncRNA biology, which may be applied to cell types and molecular processes related to type-2 inflammation.

### 2.1. Cell Type Specificity of lncRNAs

One of the most intriguing aspects of lncRNA biology is the remarkable cell type specificity of their expression and function. Recently, two groups performed unbiased CRISPR based screens and validated these claims of cell type specificity, expression and function. Liu et al. developed a comprehensive genome-wide CRISPRi library targeting the transcription start-sites of over 16,000 lncRNAs in seven distinct cell lines [75]. Remarkably, of the 499 lncRNAs identified as being necessary for cell growth, 89% modified growth in only one of the seven cell lines assayed. Similarly, in a separate study using a distinct CRISPR screening method to disrupt the function of ~ 11,000 lncRNAs in K562 cells, the authors identified 230 lncRNAs necessary for cell growth in K562s. Repeating the screen in either Hela of GM1278 cells yielded distinct subsets of essential lncRNAs for cell growth, supporting the conclusion of cell-type specific lincRNA dependency.

### 2.2. lncRNA Modes of Action: Nuclear Activity

**Figure 2:**
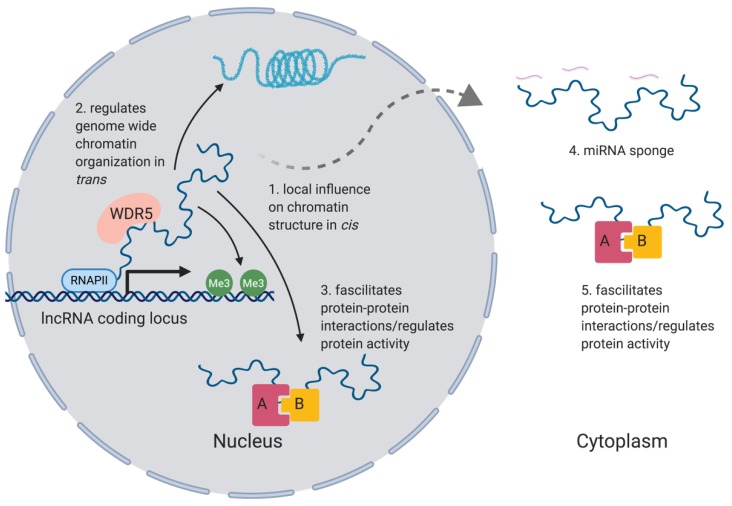
**lncRNAs elicit their activity through a variety of mechanisms.** As depicted, these may be nuclear or cytoplasmic in nature. WDR5 is shown to associate with the lncRNA as an example, but represents chromatin modifying enzymes in general. For more detailed descriptions of the molecular mechanisms by which lncRNAs function, we recommend [67].

The function of the vast majority of lncRNAs remains unknown. Cellular localization of an individual lncRNA can provide early insight into its potential mechanism of action [67]. lncRNAs (like proteins) may be nuclear, cytoplasmic or both. In the nucleus, a number of lncRNAs have been implicated in both local and large-scale chromatin organization through their ability to act as a scaffold for various transcriptional and chromatin modifiers (Figure 2). This review will highlight some key aspects of lncRNA biology, for a more detailed description please see ref: [67]

In 2010 Gupta and colleagues revealed that the lncRNA HOTAIR associated with two distinct chromatin modifying complexes, LSD1 (a demethylase) and PRC2 (a methyltransferase) and facilitated the interaction between the two complexes, coordinating their distribution on a genome-wide scale [76]. Specifically, depletion of HOTAIR resulted in a loss of both PRC2 and LSD1 from 40% of their co-occupied genes. This study was a remarkable example of a how a single lncRNA can not only influence protein-protein interactions but also function as a scaffold for multiple chromatin modifying enzymes that subsequently regulate the expression of a vast set of genes.

In the immune system, the lincRNA-EPS may function in a similar capacity [77]. lincRNA-EPS is expressed in dendritic cells and macrophages where it localizes to the nucleus and associates with chromatin. In resting macrophages lncRNA-EPS transcriptionally represses immune response genes by influencing nucleosome position and limiting chromatin accessibility at distant genes such as *Cxcl0*, *Irf7*, *Cxcl2* and others. lncRNA-EPS is down-regulated when macrophages are exposed to LPS thus permitting these genes to be transcribed. How exactly lncRNA-EPS is influencing chromatin is still unclear, but it is likely associating with specific chromatin factors/transcription factors to facilitate this activity. To this end, numerous lncRNAs have been shown to function in *cis,* influencing the expression of directly adjacent genes. A seminal study in 2016 from Engreitz and colleagues found that five lncRNA loci influenced the expression of neighboring genes. Remarkably, the lncRNA transcript itself was dispensable, but the act of transcribing the locus appears to exert an influence on the neighboring gene [68].

A remarkable example of a lncRNA that functions in this capacity, within the immune compartment of mammals is Morrbid (myeloid RNA regulator of BIM-induced death) [78]. Morrbid is a conserved lncRNA expressed in neutrophils, eosinophils and additional short-lived monocytes. In mice, loss of Morrbid leads to a significant reduction of the aforementioned cell-types, making the animals highly susceptible to bacterial infections, while protecting them from eosinophil-driven airway inflammation in response to allergens. The authors nicely demonstrated that Morrbid controls monocyte lifespan via its influence on the upstream protein-coding gene Bcl2l11, where loss of Morbid results in an increased abundance of Bcl2l11 mRNA and protein [78]. When cells received pro-survival cytokines, including IL-3, IL-5 and GM-CSF, Morrbid expression increases and Bcl2l11 levels subsequently fall. Morrbid has been proposed to function in the recruitment of PRC2 (polycomb repressive complex 2) to the Bcl2l11 locus, influencing the deposition of the repressive histone modifications H3K27me3 halting the ability of RNAPII to transcribe the locus [78].

Another lncRNA that influences the expression of its neighboring gene is PTPRE-AS1. A transcriptome-wide analysis of lncRNAs in bone marrow–derived macrophages (BMDMs) identified the lncRNA PTPRE-AS1 as being significantly induced during the IL-4 mediated maturation of M2 macrophage. [79]. PTPRE-AS1′s genomic position maps to opposite strand of the gene encoding tyrosine phosphatase receptor type E (PTPRE). To this end, loss of *PTPRE-AS1* corresponds with a significant reduction in PTPRE transcript and protein, suggesting a role for the lncRNA in promoting the expression of PTPRE in *cis*. The proposed mechanism is that PTPRE-AS1 associates with the chromatin modifying protein WDR5 and guides the deposition of H3K4me3 at the *PTPRE* promoter. In accordance with this model, reduced levels of *PTPRE-AS1* and *PTPRE* is observed in PBMCs from patients with allergic asthma [79]. Additional lncRNAs, including NIFK-AS1, have been demonstrated to influence M2 polarization of macrophages downstream of IL-4 signaling [80]. Together these studies further highlight the importance of lncRNAs in influencing/regulating cell-states in response to specific cues. Going forward, it will be interesting to determine how type-2 cytokines influences the expression (and function) of lncRNAs in distinct cell types.

Within the adaptive immune compartment, a pattern of cell type-specific LncRNA expression/function is also apparent. For example, Hu et al. examined the expression of lncRNAs in 42 subsets of thymocytes and peripheral T cells at multiple time-points during differentiation [81]. Separating T cells into various subsets, including double negative (CD4^–^CD8^–^), single positive (CD4^+^CD8^−^ or CD4^−^CD8^+^), double positive (CD4^+^CD8^+^), naive CD4^+^ T cells and T helper cells revealed that over 50% of the observed lncRNAs were stage or lineage specific (in contrast to just 6%–8% of protein coding genes). STAT4 and STAT6, transcription factors known to regulate T_H_1 and T_H_2 differentiation, respectively, were enriched at the loci encoding lncRNAs preferentially expressed in T_H_1 or T_H_2 cells. Furthermore, STAT6 was shown to be necessary for maintaining robust expression of T_H_2 specific lncRNAs. Similarly, GATA3 was also shown to promote the robust expression of T_H_2-associated lncRNAs. One such T_H_2 specific lncRNA, identified by Hu et al., was LincR-Ccr2-5′AS. LincR-Ccr2-5′AS is co-expressed with protein-coding genes associated with chemokine-mediated signaling and knockdown of LincR-Ccr2-5′AS resulted in the altered expression of a number of adjacent and distantly located protein coding genes. Specifically, depleting LincR-Ccr2-5′AS resulted in 656 and 709 mRNAs being down or up regulated, respectively. It remains unclear as to how LincR-Ccr2-5′AS regulates the expression of these target transcripts.

In human cells, RNA-seq identified ~2000 lncRNAs expressed in T_H_1, T_H_2 or T_H_17 cells subsets [82]. Of note, lncRNAs with highly lineage-restricted expression are frequently adjacent to protein-coding genes with similar patterns of expression. Specifically, 37%, 46% and 17% of lineage specific mRNAs were within 300kb of lineage specific lncRNAs in T_H_1, T_H_2 or T_H_17 cells, respectively, reinforcing the idea that some lncRNA influences the expression of neighbor genes. One novel lncRNA, T_H_2 -LCR lncRNA, is positioned in proximity to the *Il4*, *Il5* and *Il13* coding sites [82]. Knockdown of T_H_2 -LCR using siRNAs results in significant decreases in the expression of IL-4, IL-13 and IL-5. lncRNA T_H_2 -LCR is enriched in the nucleus and is necessary for maintaining the appropriate levels of H3K4me3 at the promoters of *Il4* and *Il13,* possibly by influencing the recruitment of the chromatin-modifying complex, like WDR5, to these sites [82].

Finally, GATA3-AS1 is another T_H_2 specific lncRNA that shares a promoter with the locus encoding the GATA3 transcription factor [83]. This lncRNA is restricted to the nucleus, and is necessary for the expression of *Gata3, Il5* and *Il13.* GATA3-AS1 associates with the H3K4 methlytransferase complex MLL, likely recruiting the complex to the *Gata3* locus to promote a chromatin environment that is permissive to active transcription [84].

### 2.3. lncRNA Cytoplasmic Activity

An increasing amount of evidence indicates that transcription factors (TFs) interact directly with RNA and that these interactions may be regulatory in nature. An early study in 2005 performed an shRNA screen targeting ~500 conserved ncRNAs in Hek293 cells [85]. This study identified a ncRNA (now called NRON noncoding repressor of NFAT) that functions to repress Nuclear Factor of Activated T cells (NFAT), which is activated early in T_H_2 cell differentiation. NRON associated with nuclear transport proteins and an increase nuclear localization of NFAT. Whether NRON regulates T_H_2 cell differentiation or not remains to be determined.

Similarly, lncRNA-DC was identified as being robustly expressed in Dendritic Cells (DCs), and knock-down results in a failure of DC differentiation. Remarkably, lncRNA-DC localizes in the cytoplasm where it associates with STAT3, blocking STAT3 dephosphorylation by SHP1, thus keeping STAT3 active, and permitting it to enter the nucleus [86].

Taken together, these examples highlight the importance of lncRNAs in contributing to immune cell differentiation and function in a variety of different cell types, including those implicated in type-2 immunity. The observation that lncRNAs display cell-type specific expression profiles, yet influence the activity of more broadly expressed proteins makes them attractive therapeutic targets, with potentially less unintended toxicity. That said, to date, designing molecules that specifically target RNA has remained tenuous. Going forward, developing strategies to robustly target lncRNAs will be necessary before they can entertain as robust therapeutic targets.

## 3. MicroRNAs in Type-2 Immunity

### 3.1. miRNA Biogenesis

MicroRNAs (miRNAs) are single stranded, 19-22nt long RNAs found in metazoans, plants and in some viruses, and regulate gene expression post-transcriptionally. Typically, a miRNA engages a target mRNA at its 3′ UTR, following Watson–Crick nucleotide pairing, to initiate target degradation and/or inhibition of translation. Humans have ~1900 annotated miRNA genes [87], some evolutionary conserved, others more recently acquired. Let-7, for example, is a miRNA family discovered in *C. elegans* that had remained practically unaltered throughout evolution, with demonstrated implications in tissue differentiation and tumor suppression [88].

**Figure 3:**
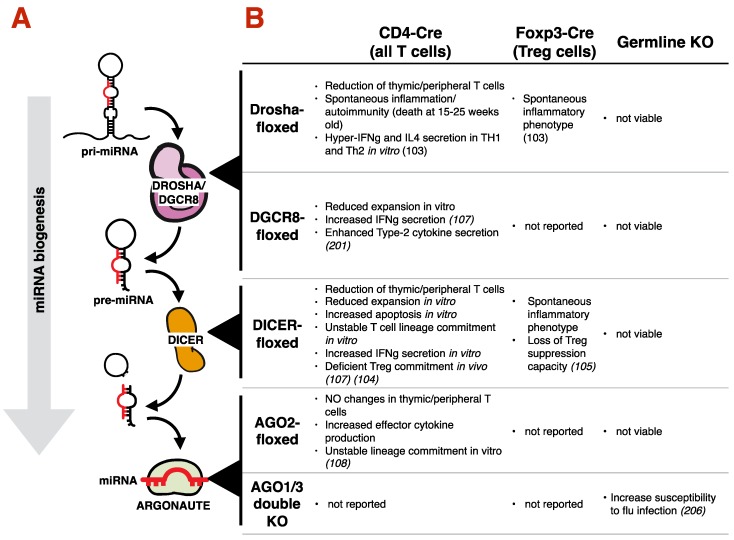
miRNA biogenesis and its impact on T cells function and Type2 immunity. (**A**) An immature RNA strand that contains a typical stem-loop structure (pri-miRNAs) is transcribed by RNA polymerase-II, and subsequently recognized by the RNAse DROSHA, in complex with DGCR8. DROSHA cleaves the pri-miRNA to produce a shorter, pre-miRNA, which is in turn the substrate of the RNase DICER, the enzyme that removes the loop from the structure. A single strand of RNA is then loaded on an Argonaute protein, to produce a mature miRNA. (**B**) A summary table describes the impact that proteins involved in the maturation miRNAs has on T cells and adaptive immunity. The first column indicates the floxed gene. The first row indicates the promoter (and the cell type) that drives Cre expression that lead to gene deletion in a specific cell subset in mice.

**Figure 4:**
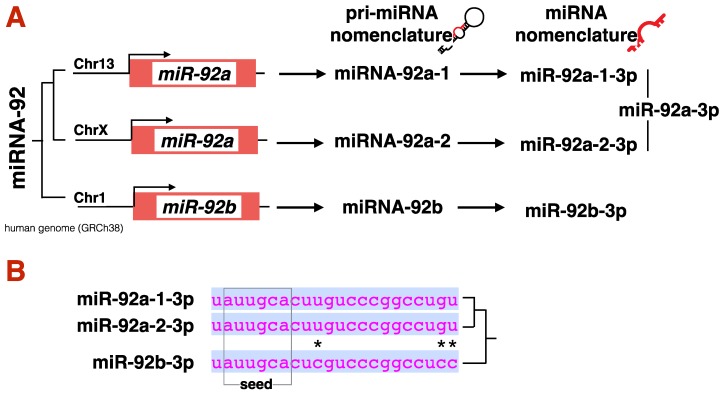
**miRNA complete nomenclature**. A layer of complexity in the miRNA field is the fact that some miRNA genes have paralogues, thus the same miRNA may arise from two different location in the genome. When a miRNA is encoded by two or more genes (as in example miR-92), the complete nomenclature should include a number that refer to the locus-of-origin of such miRNA, i.e., miR-92a (which share the same seed sequence with miR-92b, yet they are not identical (**B**)), is produced by two genes in humans, in chromosome 12 and chromosome X (**A**). The complete nomenclature for miR-92a thus includes a “-N” to indicate the distinct origin of the pri-miRNA (miR-92a-1 (from Chr 13) and miR-92a-2 (from Chr X)). This system is particularly necessary in experiments where pri-miRNAs are reintroduced in cells using vectors or synthetic RNA, as distinct pri-miRNAs may be processed differently by DICER. However, while the sequence of the pri-miRNA stem-loop between miR-92a-1 and miR-92a-2 is distinct, the resulting mature miRNA is in actuality identical. Although from different loci, the origin of the mature miR-92a-3p in cells is the result of the transcription of two genes, and only genetic testing with individual gene KO may reveal the contribution of each locus to the final miRNA pool. When we refer to a mature miRNA with district gene origin, we drop the “-N” nomenclature and simply refer to it as miR-92a-3p (A—far right). (*) marks diverse nucleotides between miR-92a-3p and miR-92b-3p

In the canonical miRNA biogenesis pathway (Figure 3A), genes that encode a miRNA are transcribed by RNA Polymerase-II (RNAPII) to produce a long precursor-miRNA (pri-miRNA) with a hairpin structure. Pri-miRNAs are recognized by RNAses in the nucleus (DROSHA) and cytoplasm (DICER) to produce a short, double-stranded RNA with overhanging ends. Only one of the two RNA strands will be loaded onto an Argonaute (AGO) protein, to become a functional miRNA. The complete nomenclature for any miRNA includes the name of the miRNA gene it comes from, followed by a -3p or -5p depending on the strand from which it is derived from. As in most cases a mature miRNA is the result of only one of the two arms of the RNA-hairpin, most authors avoid complete nomenclature. For example: miRNA-155 is derived from the 5′-end of its pre-miRNA. Thus, the complete nomenclature should be miR-155-5p, yet it is often just referred in literature as simply miR-155). To avoid confusion, this review will stick to complete nomenclature. For further clarification on miRNA nomenclature, refer to Figure 4. For a detailed description of miRNA biogenesis, including non-canonical miRNA biogenesis, we recommend other readings [89].

Once a miRNA is loaded onto a AGO protein, it can target mRNAs in the cytoplasm, where it initiates the recruitment of the remaining components of the RNA Induced Silencing Complex (RISC) [90]. GW182 is a conserved, AGO-interacting protein, which plays a central role in RISC. GW182 bridges AGO proteins to other RISC proteins, including the 5′ de-capping deadenylases and exonucleases enzymes, that collectively degrade the target RNA [91,92]. Mammals have four AGO proteins (AGO1, AGO2, AGO3 and AGO4) that bind to miRNAs, all of which are thought to bind to the same miRNA repertoire [93], and potentially form a RISC [94,95]. In spite of substantial structural and expression similarities across AGO isoforms in cells and tissues [96], these proteins are not redundant. AGO2 is the only essential AGO for life in mammals [93]. AGO2 is does more than simply bind mature miRNAs, as it actually supports the DICER-dependent miRNA maturation process [93,97]. AGO2 is also the only ARGONAUTE with a catalytically active endonuclease pocket, able to cleave complementary RNA [93] (a recent study found that AGO3 can also carry slicing function, although only in special circumstances [98])

As we describe the function of miRNAs in type-2 immunity, it is important to note that in animals miRNA-mRNA pairing is a dynamic process with a variety of regulatory features, including i) the local RNA secondary structure [99], ii) the post-transcriptional modification of the AGO protein [100] and iii), and the extent of base pairing between miRNA and target [101]. The ‘seed sequence’ of a miRNA is a 7–8 nt region at the 5′-end of the miRNA that best predicts the likelihood of a miRNA to target a mRNA (Figure 4B). The rest of the miRNA sequence does not have to be complementary to the target mRNA to render an RNA targetable, although McGeary and colleagues recently showed that, in certain circumstances, a miRNAs can bind to mRNA independently of seed-matching [102]. Regardless of the molecular mechanism, the strength by which a miRNA binds to a target mRNA dictates its fate, directing it to degradation (strong binding/high complementarity) or to simply halting the mRNA translation and preserving the mRNA molecule (weak binding/low complementarity) [103]. This lax rule for miRNA-mRNA paring makes true miRNA targets hard to predict in silico [104], and reveals that most miRNAs have a small effect on a large number of targets [105]. Although the majority of the literature presented here describe a single miRNA-mRNA pair as ‘the’ molecular mechanism behind a specific type-2 immune response, these authors are caution on such direct-line conclusions, and prefer to emphasize a model in which a miRNA has a modest effect on numerous genes that collectively influence immunity.

### 3.2. Requirement of the miRNA Machinery for T Cell Development, Differentiation and Type-2 Immunity

Several studies have investigated the broad role of miRNAs in T cells and type-2 immunity (Figure 3B). Disrupting essential components of the miRNA machinery, including *Dicer*, *Drosha* or *Dgcr8*, has revealed that miRNAs are essential for the immune compartment. Blocking miRNA biogenesis in CD4^+^CD8^+^ double positive thymocytes (the developmental stage before CD4^+^ and CD8^+^ T cell maturation) results in a reduction in peripheral T cells in mice [106,107]. Furthermore, a spontaneous hyper-inflammatory phenotype and premature death at 15–25 weeks age is also observed in these mice, in part, driven by dysfunctional T_REG_ cells [106,108,109]. In vitro, T helper cells with compromised miRNA biogenesis (such as CD4-Cre Drosha-f/f; CD4-Cre Dgcr8-f/f and CD4-Cre Dicer-f/f T cells) are hyper-reactive, divide quickly, and secrete more cytokines then WT controls [106,107]. Interestingly, this hyper-reactivity also leads to a more rapid collapse and contraction of the T cell pool, helping to explain the reduced number of peripheral T cell in vivo [106,107]. Using similar genetic tools, several investigators found defects in T cell commitment to a specific lineage in the absence of miRNAs (for example: T_H_2 cells produce both T_H_2-associated IL-4 and T_H_1-associated IFNγ), suggesting that miRNAs contribute to T cell development, differentiation, lineage commitment and effector function [107,110,111,112].

### 3.3. Impact of Specific miRNAs on Type-2 Immunity

In this section of the review, we will look at miRNAs found in tissues or specific cells present in patients or in animal models of type-2 disease (including allergic dermatitis and asthma), elaborate on their mechanisms of action, and identify potentials for therapeutic interventions.

Allergic asthma, characterized by elevated airway eosinophilia, mucus hypersecretion in the lung, and elevated circulating IgE, is a classic type-2 immune-mediated disease [113]. Asthmatic patients have a remarkably altered miRNA signature in their lung epithelial cells compared to healthy controls [114]. Whether this is due to the tissue response, immune cell activation or therapeutic intervention—such as the use of inhaled corticosteroid (ICS)—is unclear and warrants further investigation [114,115]. Efforts to untangle these factors have employed in vitro models of primary human bronchial airway epithelial cells (HBEC). Stimulation of HBEC from healthy donors with the type-2 cytokine IL-13 induced a similar miRNA signature as that seen in lung epithelial brushings taken from asthmatic patients, supporting the notion that a type-2 miRNA signature may indeed be attributable to type-2 biology acting on stromal cells [114].

Similarly, miRNA profiles have been described in atopic dermatitis (AD), in which the orchestration of epithelium, myeloid and lymphoid cells contribute to the establishment and maintenance of a type-2 inflammatory state in the skin. Global miRNA expression profiling in healthy and lesioned skin of patients with atopic dermatitis found the differential expression of a suite of miRNAs [116], including -3p and -5p arms of miR-501, miR-223, miR-155, miR-31 and miR-187. Using a chemically-induced allergic reaction, Vennegaard et al. identified a miRNA signature in allergic contact dermatitis that was similar in both humans and mice [117]. miR-223, miR-21, miR-146b, miR-142, were consistently upregulated in skin of both species 48h after allergen challenge. Small-RNA sequencing of psoriatic skin also revealed keratinocyte specific miRNA dysregulation in this inflammatory disease, with dysregulated miR-31, miR-206, miR-21 and miR-135b (10.1093/hmg/ddr331). A comprehensive look at the altered miRNAs in type-2 human and mouse diseases will facilitate the generation of a roadmap and strengthen the case for further investigations (Table 1).

To date, there are no studies to have profiled small-RNAs in whole human or animal tissues during parasitic helminth infections, which are controlled by type-2 immune response, representing an area needing more exploration. However, our laboratory has profiled the miRNA repertoires of T cells isolated from parasite infections in mice, and identified miRNAs that controls T cell effector function and type-2 immunity. For example, the Th2-associated Treg cells (*Foxp3*-reporter positive) (isolated from *Schistosoma mansoni* infected mice) upregulate miR-182-5p, which functionally mitigate IL-2 secretion through the repression of *Il2*-promoting genes, thus contributing to the Treg-curtail of the type-2 immune response [118]. Similarly, miR-15a-5p, miR-20b-5p, miR-146a-5p, miR-155-5p and miR-200c-3p are all differentially expressed in Th2 cells (*Il4*-reporter positive) isolated from *Heligmosomoides polygyrus* infected mice, with miR-155-5p bearing a functional impact in such model. Those differentially expressed miRNAs were also found in Th2 cells isolated from the house dust mite asthma model, and in vitro differentiated Th2 cells [119].

Using Table 1 as guidance, we will explore Type2-associated miRNAs, discuss their targets and asses their therapeutic potential.

#### 3.3.1. miR-34/449

**Figure 5:**
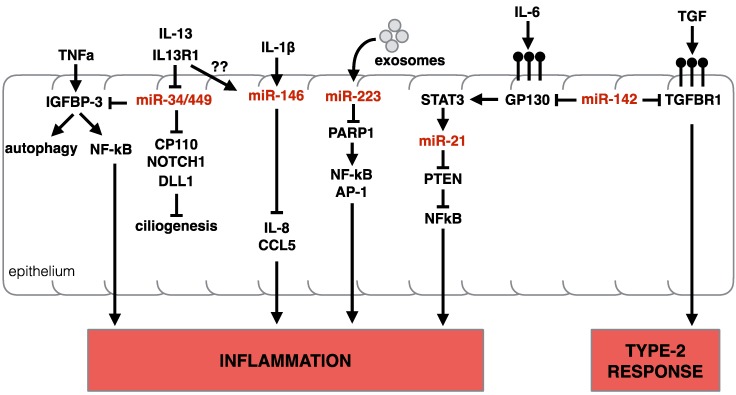
**Type-2 related miRNAs in epithelial cells.** Epithelial cells represent the first barrier to respond to stimulation and insult from the environment. In type-2 immunity, epithelial cells in mucosal barriers are particularly relevant and essential players in the initiation of an immune response.

In animals, the miR-34/449 family consists of six homologous miRNAs (Chr1: miR-34a-5p Chr1: miR-34b-5p, miR-34c-5p; and Chr5: miR-449a-5p, miR-449b-5p, miR-449c-5p), encoded in three genomic loci. miRNAs belong to the same family when their ‘seed regions’ are identical (see above). This suggests these miRNAs also share similar targets. Functionally, miRNAs in the miR-34/449 family regulate *Cp110* (a centriolar protein that suppresses cilia assembly [120]), *Notch1,* and *Dll1* (genes with a role in airway metaplasia and mucous clearance in asthma [121,122]). Noteworthy, miR-449a/b/c are highly enriched in multi-ciliated cells of the human airway epithelium [123]. miR-34/449 family also target *Igfbp-3*, a transcript with an established role in modulating TNF-α induced expression of NF-κB [124], and recently suggested to modulate autophagy in the airway epithelium [125], possibly contributing to the pathogenesis of asthma. Due to its central role the homeostasis and differentiation of the epithelium, miR-449a/b/c knockdown results in the suppression of multiciliogenesis [123], and mice deficient for all three genomic loci of the miR-34/449 family die post-natal due to defective mucociliary clearance of the airways [120]. IL-13 signaling in human lung airway epithelial cell line BEAS-2B [125], or in primary human basal epithelial cells [114] caused a decrease in miR-34 and miR-449 expression as early as 3 days post exposure, consistent with the cytokine role in shaping cellular composition, reducing ciliated cells and promoting goblet cell hyperplasia (in Solberg et al. microarray could not differentiate between miRNA isoforms). It is plausible that the downregulation of miR-34/449 by IL-13 contributes significantly to the pathogenic functions of IL-13. Although the precise molecular mechanisms by which IL-13 modulates this family of miRNAs is unclear, it is tempting to speculate that this represents an opening for therapeutically intervention in allergic asthma [125]. A summary of miR-34 and miR-449 activity in epithelial cells (along with other miRNAs) is presented in Figure 5.

#### 3.3.2. miR-223

miR-223-3p is upregulated in the skin of individuals with atopic dermatitis [116,117]. miR-223-3p is also upregulated in a murine dermatitis skin model [117], and in tissue from eosinophilic eosophagitis (EoE) patients [126], making this miRNA an interesting target for the control of type-2 immunity. miR-223-3p is highly expressed in the myeloid compartment of the immune system, and was first described as a regulator of stem cell/progenitor cell proliferation, granulocyte differentiation and granulocyte activation in mice [127]. In common-myeloid progenitor cells (CMPs), miR-223-3p targets *Mef2c*, a transcription factor that promotes myeloid progenitor proliferation, to control the number of progenitors in the bone marrow [127].

**Figure 6:**
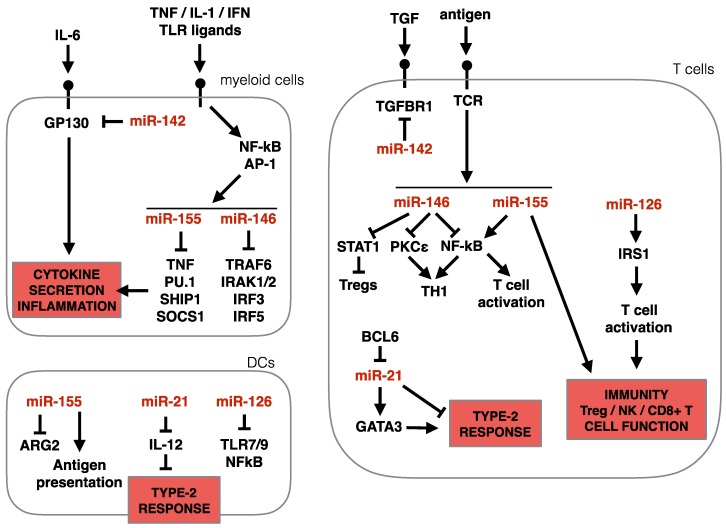
**Type-2 related miRNAs in the hematopoietic compartment.** Blood cells that play a significant role in the type-2 immunity are controlled by type-2 related miRNAs.

In the periphery, miR-223-3p suppresses neutrophil [127] and eosinophil [128] maturation, granulocyte activation and their responsiveness to stimuli. Mechanistically, in eosinophils, miR-233-3p targets the insulin-like growth factor 1 Receptor (*Igf1r*), a hormone that stimulates cell proliferation and inhibits cell death [128], thus curtailing eosinophil responses. In macrophages, miR-223-3p can promote alternative activation (M2) via the inhibition of *Pknox1*, which instead supports canonical (M1) activation [129]. Alternative activation of macrophages is induced via IL4Rα-signaling, a pathway common to both IL4 and IL13. Alternatively, activated macrophages are present in many type-2 disease settings, wound healing processes and cancer. In the context of allergic asthma, inhibition of M2 polarization via cynaropicrin, a galectin-3 pathway inhibitor, led to a reduced asthmatic response, reduced eosinophilic inflammation and reduced collagen deposition in the airways in a mouse model [130]. If repeatable and translatable, such findings would spark more interest in reduction of the M2 polarization response in type-2 diseases, making the delivery of miR-223-3p an interesting therapeutic option. In addition to suppressing M2 polarization, delivered miR-223-3p may curtail type-2 inflammation via neutrophil and eosinophil suppression [131]. Although overlooked for years, the miR-223-3p -mediated regulation of stromal cells has recently been appreciated, with one study finding that miR-223-3p targets NF-κβ in epithelial cells of zebrafish [132], further contributing to the immune modulating functions of miR-223-3p. Finally, it is worth noting that an extracellular role for miR-223-3p has also been described. Produced by neutrophils, miR-223-3p was found to be transferred, via extracellular vesicles, to lung epithelial cells in vitro [133]. In epithelial cells, miR-223-3p targets *Parp1*, a gene that can facilitate inflammatory responses via activation of NF-κβ and AP-1 [133]. Thus, the presence of increased levels of miR-223-3p in tissue biopsies of patients with type-2 associated diseases may be reflective of the influx of miR-223-3p expressing myeloid cells or the transfer of miR-223-3p, via extracellular vesicles, to epithelial cells, or a direct dysregulation of this miRNA in the stroma. A summary of miR-223-3p activity in epithelial and blood cells (along with other miRNAs) is presented in Figure 6.

#### 3.3.3. miR-142

miR-142 is also consistently upregulated in biopsies of allergic dermatitis patients [116,117], in esophageal tissue of EoE patients [126] and in an IL-13-transgenic allergic mouse model [134]. Intriguingly, both -5p and -3p mature miRNAs are detectable for miR-142. miR-142 is a rare example where both strands of the pri-miRNA appear to be functional in cells [135]. In general, miR142-5p is observed at higher levels than the -5p arm (*84*), but whether the ratio between the two miRNAs is relevant in immunity is unknown, and most investigations using knock-out mice concomitantly eliminate both -5p and -3p miRNAs. Both miR-142-3p and miR-142-5p are abundant in cells of hematopoietic origin, and play a role in lineage differentiation of hematopoietic cells. Both can target *Il6st*, a gene encoding for Gp130 [136], the co-receptor protein for the IL-6 cytokine family. IL-6 is found in sputum of asthmatic patients [137] where it plays multiple roles, like enhancing IL-4 secretion from T_H_2 cells, and promoting T_H_17 differentiation [138]. miR-142-3p and -5p have a strong effect on immunity in multiple diseases as they target nodal genes of several immune response pathways like *Stat1a* in Neutrophils, *lpp* in Mast cells, and *Socs1* in macrophages [136]. Consistent with this, miR142-deficient mice develop an immune proliferative phenotype, accompanied by adaptive immunodeficiency with reduced T and B cells [139]. A closer look into type-2 immunity, miR-142-3p was found in bronchial biopsy in asthmatic humans and in OVA-inflamed mouse lungs [140]. The role of miR-142-3p in asthmatic or type-2 dermatitis models is not reported, and considering its pleiotropic effect on several immune-regulatory genes, it is difficult to predict its function in this pathology. A speculation is drawn by the fact that miR-142-3p targets *Tgfbr1*, part of the TGF-b superfamily receptor complex, a cytokine that support M2 macrophages and T_H_17 differentiation. As mentioned above, there is evidence for the pathogenic role of M2 macrophages in allergic asthma. As such, over-expression of miR-142-3p in M2 macrophages, but not M1 macrophages, induced apoptosis via suppression of Tgfbr1 axis [141].

#### 3.3.4. miR-146 and miR-155

miR-146 and miR-155 are two well-studied miRNAs with a widely reported influence on the immune system. Similar to miR-142, miR-146 and miR-155 interfere many immune-relevant pathways, yet only one arm of the pri-miRNA give rise to a functional miRNA. In the context of type-2 immunity, miR-146a-5p and miR-146b-5p and miR-155-5p are found upregulated in the tissue of atopic dermatitis patients and in a variety of pre-clinical allergy models, EoE esophagus, and allergic asthmatic lungs (see Table 1). Expression of these miRNAs are tightly linked to the presence of pro-inflammatory stimuli, such as TNFα, IL-1β, IFNs and TLR ligands, particularly in myeloid cells. Pre-miR-155 and pre-miR-146a are both transcribed by the NF-κβ transcription factors, with evidence that miR-155 is additionally controlled by Activator Protein 1 (AP-1) [142,143], explaining the pleiotropic expression of these miRNAs following the aforementioned stimulations. In lymphocytes, both miRNAs are upregulated following BCR and TCR stimulation [144]. These miRNAs repress several TLR4 effector proteins, such as TNFα, PU.1, SHIP1, SOCS1 (miR-155), and TNFR-associated factor 6 (TRAF6), IRAK1, IRAK2, IRF3 and IRF5 (miR-146a) [144]. Similar to miR-142-3 and -5p studies, mice deficient for either miR-146 or miR-155 genes have illustrated their importance in the regulation of the immune system. Both miR-146a-/- and miR-155-/- mice suffer from different forms of autoimmunity [145,146]. Aging miR-146a-/- mice develop splenomegaly, lymphadenopathy, and die prematurely with multiorgan inflammation manifested by lymphocytic and monocytic infiltration in the liver, kidneys, and lungs, with some evidence of tissue damage. Aging miR-155-/- mice develop a more restricted autoimmunity to just the lung tissue: aged mice display significant remodeling of lung airways, with increased bronchiolar subepithelial collagen deposition and increased number of leukocytes in bronchoalveolar lavage fluids [146]. MiR-155-/- mice also develop spontaneous enteric inflammation at a younger age [146]. These features awarded miR-155 and miR-146 the status of pan-suppressors of the immune system.

miR-146-a and miR-146-b share identical seed regions and are thought to target the same mRNA. miR-146a is more abundantly observed in small-RNA-Seq data from numerous sources, but both isoforms are upregulated in response to stimuli. Interestingly, during eosinophilic differentiation from bone marrow precursors in vitro, miR-146a decreases while miR-146b increases [147] hinting to a possible non-redundant function of these two miRNAs in a type-2 relevant myeloid cell, however the function of these miRNAs in eosinophil development and function is unknown.

In the monocyte cell line THP-1, over-expressing miR-146a dampens the response to LPS stimulation, and reduces the secretion of TNFα and IL-6 [145]. In T cells, miR-146a also suppressed TCR-mediated activation, by targeting the NF-κβ pathway [145,148], and by reducing its own expression level via a feed-back loop mechanism [142,143]. Among the T helper cell subsets, miR-146a seems to preferentially influence T_H_1 cells, where it is more highly expressed, relative to other T_H_ cells. In T_H_1 cells, miR-146a regulates Protein Kinase Cε (PKCε), which forms a complex with STAT4 to drive T_H_1 cell differentiation in response to IL-12 [149]. miR-146a also helps to maintain T_REG_ cell identity via the direct suppression of *Stat1* [150]. Our lab found miR-146a consistently upregulated in in vitro polarized and ex vivo isolated T_H_2 cells, suggesting a role for this miRNA in T_H_2 cells. Using bone marrow chimeric models, in which miR-146a-deficiency was restricted to the T cell compartment, we found that miR-146a was indeed required to dampen T-cell driven lung inflammation in a HDM model [109], consistent with the published role of miR-146a as an immune activation modulator. We also discovered that miR-146a is necessary for the commitment of T cells to a T_H_2 polarization program, as miR-146a-/- T cells led to a mixed response in HDM-challenged lungs, and were unable to mount a competent T_H_2 response to expel the intestinal helminth, *Trichuris muris*, a parasite that requires the full engagement of the type-2 immune response [109]. Although miR-146a has primarily been studied within the immune compartment, new evidence reveals its role in stromal cells, including human lung alveolar epithelial cells, which respond to IL-1β stimulation by up-regulating miR-146a, that in turn suppresses IL-8 and CCL5 chemokines through the direct target of the 3′ UTRs of these mRNAs [151].

The miR-155 gene locus is highly conserved, and in humans lies within the third exon of BIC (B cell integration cluster). miR-155-5p is a known oncogene in B cell lymphomas, as it regulates a number of genes that suppress B cell proliferation and survival. These targets include transcription regulators, receptors, and signaling pathway components, e.g., HDAC4, PIK3R1, SMAD5, SHIP1, PU.1, BCL2, and C/EBPβ [152]. miR-155 also regulates inflammation by regulating members of the TNF-receptor superfamily, such as TNF and TRAMP (*Tnfrsf25*) [153,154], and importantly via the suppression of *Sfpi1*, the gene encoding for PU1. PU1 is a dose-sensitive transcription factor [155], that regulates gene expression in many cell lineages [156,157], controls hematopoiesis [158,159,160] and is a signature transcription factor for maintenance of myeloid, dendritic, and B cells [161,162]. In differentiated lineages, PU.1 is a negative regulator of class-switch-recombination [163] and terminal B cell differentiation [164]. In T cells, PU.1 controls thymocyte developmental progression [165] and negatively regulates GATA-3 activity [166]. Despite early evidence suggesting an immune-suppressing function for miR-155, the majority of the experimental evidence show that miR-155 is actually necessary for the establishment of a functional immune response, including a type-2 immune responses [119], and autoimmune response [167,168]. In particular, our lab found that miR-155-/- mice are resistant to HDM-induced airway inflammation and lung remodeling. Eosinophilia, and the induction of the IL-13-mucus secretion axis in the lung was also dependent on this miRNA. As miR-155 was found strongly upregulated in T_H_2 cells and elevated in lung tissue of asthma models, our data suggest a role for miR-155 in T_H_2-mediated airway allergy [119], consistent with others [169].

It is worth noting that the role for miR-155 as a master regulator of inflammation extends beyond T_H_2 cells, miR-155 is necessary for T cell activation, polarization and T-helper mediated B cell class-switching/maturation [146]. The ability of miR155 to influence T cell polarization and effector function has been reliably demonstrated by multiple investigators, with all data indicating that miR-155 is required for all types of T cell differentiation [119,146,170,171]. miR-155 was recently found to directly target *Socs1*. SOCS1 protein binds JAKs proteins, and is involved in the negative regulation of cytokines that signal through the JAK/STAT3 pathway. The introduction of point mutations at the 3′-UTR of *Socs1* (where miR-155 binds) by Lu et al. elegantly demonstrated that a miR-155: *Socs1* axis is required for T_REG_ cell fitness and NK and CD8^+^ T cell function during viral infection [172]. miR-155 appears to be broadly required for immunity against a variety of pathogens, including bacterial, viral and parasitic helminths [109,146,172], thus influencing immunity from a broader angle, making this miRNA an interesting target for therapeutic intervention in a variety of hyper-inflammatory diseases. Beyond T cells, miR-155 directly controls Arginase 2 (*Arg2*) and *purinergic receptor* signaling in dendritic cells (DC), influencing the DC phenotype: *Arginase 2* and *purinergic receptor* are both required for optimal activation of T-cell responses [173,174]. miR-155 is also required for antigen presentation in DCs [146]. Group 2 innate lymphoid cells, (ILC2) also upregulate miR-155 in response to stimulation (IL-33), and need miR-155 for optimal expansion and IL13 secretion in vivo [169]. In ILC2, miR-155 also targets *Socs1*, a known regulator of ILC2 biology [175].

#### 3.3.5. miR-21

miR-21 is upregulated in the skin of allergic dermatitis patients, in the airway epithelial brushings of allergic asthmatic patients, and in a variety of pre-clinical disease models (Table 1). In mice, miR-21 is upregulated in airway tissue following a single dose inhalation of HDM [176] or during chronic OVA intra-nasal exposure [177]. Similarly, other models of type-2 inflammation, such as intra-tracheal delivery of IL-13 or lung epithelial over-expression of IL-13 or IL-4, consistently show an upregulation of miR-21 [134], in an IL-13Rα1-dependent manner. However, in allergen-driven asthma models (HDM or OVA delivery i.t.), miR-21 upregulation was IL-13Rα1 and STAT6-independent [134], suggesting that multiple pathways can drive miR-21 expression in type-2 diseases.

In human bronchial epithelial cells, IL-13 could not directly induce miR-21 [114]. Although the cellular source and regulation of miR-21 in humans remains unclear, these data identify miR-21 as a commonly found miRNA in the context of type-2 immunity. miR-21-/- mice are protected from allergen-induced airway inflammation, with reduced airway eosinophilia, and a broadly reprogrammed lung tissue transcriptome skewed towards type-1/IFNγ-driven inflammation [178]. In situ hybridization experiments of asthmatic mouse lungs identified interstitial macrophages and dendritic cells as a primary source of miR-21 expression [134]. Mechanistically, miR-21 controls the type-1/type-2 balance in multiple ways. IL-12, a cytokine driving T_H_1 cell differentiation, is a direct target of miR-21 in DCs [134]. Indeed, miR-21-/- DC produce more IL-12 compared to WT DCs [178], therefore favoring T_H_1 differentiation [179]. However, while antagonizing Th2 cell polarization, miR-21 appears also to support T_H_2 cell stability, as miR-21-regulated *Spry1*, a negative regulator of the MAP kinase pathway [180], supports GATA3 protein stabilization [181]. Finally, the transcription factor BCL6, which is essential for follicular helper T cells [182], competes with STAT3 for the binding of the miR-21 promoter region. While STAT3 promotes miR-21 expression, BCL6 suppresses it [180] thus placing this miRNA at an important junction in the regulation of T_FH_/T_EFF_ cell balance. Taken together, miR-21 represents a promising target to modulate type-2-associated airway inflammation, with pre-clinical studies demonstrating that inhibition of miR-21 can reduce airway inflammation in some, but not all settings [177,183].

#### 3.3.6. miR-126

Although there are two annotated miR-126 genes in mice (miR-126a and miR-126b from the same cluster), deposited sequencing data indicates that the miR-126b gene is virtually inactive, and it is actually absent in the human genome. The literature often refers to miR-126 or miR-126a interchangeably. The intra-tracheal delivery of HDM in mice leads to the upregulation of miR-126a-3p in airway epithelial cells within 24h [176]. Similarly, epithelial brushings from asthmatic patients have upregulated expression of miR-126-3p [115], making this miRNA of interest in type-2 immunity. The inhibition of miR-126a-3p, using synthetic oligos to antagonize miR-126-3p binding, reduces airway resistance, BAL cell infiltrate, goblet cell hyperplasia and T_H_2 cell activity in local draining lymph nodes [176], with similar results observed in an OVA-induced asthma model [177]. These observations support the therapeutic potential of targeting miR-126-3p. The precise mechanism action of hsa-miR-126-3p/mmu-miR-126a-3p in type-2 immunity is unclear, but several miR-126-regulated pathways in a variety of disease-relevant cells have been described. miR-126a-3p is highly expressed in endothelial cells, in which it controls angiogenesis [184] potentially playing a role in the tissue remodeling events seen in asthmatic patients. Within the immune compartments, miR-126a-3p controls the renewal of hematopoietic stem cells [185] and the B cell expansion and proliferation via targeting *early B-cell factor 1* (*Ebf1*) [186]. miR-126a-3p is also expressed in plasmacytoid dendritic cells (pDCs), where it promotes cell survival and type-1 interferon production in response to viral infection. In pDCs, miR-126-3p targets a number of genes involved in innate immune response, including *Tlr7*, *Tlr9* and *Nfkb1*. It is unlikely that the effect seen with miR-126a-3p inhibition on the allergic asthma models is due to its direct effect on T cell, where inhibition of miR-126a-3p enhances, rather than inhibit, T cell effector function. In T cells, miR-126a-3p directly targets insulin receptor substrate-1 (*Irs1*) [187], a gene involved in the MAP kinase pathways (pAKT and pERK signaling), which plays a key role in T cell activation. miR-126-3p prevents excessive activation, proliferation and IFNγ secretion when T cells are stimulated in vitro or in vivo [188]. miR-126a-3p also promotes the differentiation of Foxp3^+^ T regulatory cells by suppressing the same IRS1/pAKT pathway, which can hamper T_REG_ development [189]. Accordingly, miR-126-deficient mice have reduced T_REG_ cells and increased activated peripheral CD4^+^ T cells, and develop severe colitis when challenged with DSS [188]. Taken together, hsa-miR-126-3p/mmu-miR-126a-3p appears to have multiple functions in a variety of cells involved in type-2 immunity, regulating both innate, adaptive and stromal responses, in both a positive and negative manner.

#### 3.3.7. miR-375

The miRNA repertoire of the gastrointestinal tract is broadly similar between the large and small intestine, in whole epithelium, in enteroendocrine cells and in Lgr-5^+^ stem cells [190]. miR-375-3p is an abundantly expressed miRNA throughout the intestinal tract, and it appears to play a role in regulating cellular differentiation [190,191]. miR-375-3p targets *Klf5*, an antagonist of the transcription factor KLF4, required for goblet cell differentiation [192]. Interestingly, IL-13 (which induces goblet cell hyperplasia in both lung and intestine), also induces miR-375-3p expression in intestinal epithelial cell lines, which in turn releases the break on KLF4 to promote goblet cell differentiation. In accordance to this mechanism of action, mice lacking miR-375-3p have reduced expression of *Gob5* and *Relmβ* in the intestine following helminth infection, supporting the requirement of miR-375-3p for goblet cell hyperplasia and anti-helminth immunity [191]. A more complex picture emerges from a type-2 disease of the airways. Opposite to what happens in the intestine, miR-375-3p is down-regulated in the lung of an IL-13 lung-transgenic mouse model, and is down-regulated in patients with eosinophilic esophagitis (EE), compared to healthy volunteers [193]. Furthermore, human esophageal squamous and bronchial epithelial cells, exposed to IL-13 also reduce the level of miR-375-3p [193]. The direct implication of epithelial miR-375-3p in allergic asthma is unknown, as well as the exact mechanism of action by which IL-13 modulates miR-375-3p, and miR-375-3p relevant targets in the lung. Similar to miR-449(a/b/c)-5p, miR-375-3p is downstream of the IL-13 signaling axis that control goblet cell function and mucous secretion, making this miRNA an interesting target for therapeutic intervention, albeit further investigation on its role in other cells is needed.

#### 3.3.8. miR-17~92 Cluster

To identify miRNAs highly expressed in lymphocytes of asthmatic patients, Simpson et al. isolated CD4^+^ T cells from the bronchoalveolar lavage (BAL) of healthy volunteers, steroid-naive and steroid-treated asthmatic subjects [194] and assessed their small-RNA repertoire. They found that miR-19a-3p was elevated in CD4^+^ T cells of asthmatic individuals, independent of corticosteroid usage. miR-19a-3p is part of a cluster of miRNAs on human Chromosome 13. Unlike miRNA families, which share the same seed sequence, a miRNA cluster is a group of different RNA molecules under the control of the same promoter. A single RNA transcript can give rise to multiple hairpin-like structures, for which the DROSHA enzymes excise multiple pri-miRNAs, that are exported to the cytoplasm. The cluster in which miR-19a-3p resides is referred to as miRNA cluster 1 (miR-C1), which also includes miR-17, miR-18 and miR-92 genes.

T cell-specific deletion and over-expression of miRC-1 in mice demonstrated that miRC-1 is required for T_H_2 cell proliferation and for IL-4, IL-5 and IL-13 production [110,194]. Although the re-introduction of individual miRNAs from cluster-1 in miRC-1 deficient T cells could each partially rescue cytokine secretion from T_H_2 cells, miR-19a-3p outperformed the others [194]. *Pten*, *Socs1* and *Tnfair3* were found to be targets of both miR-19a-3p and miR-19b-3p (products of a separate miRNA gene). The suppression of these transcripts was necessary for IL-4 and IL-13 production in T_H_2 cells [194]. MiRC-1 miRNAs are also known to play multiple functions in T cell biology beyond T_H_2 cells. For example, this cluster is found amplified in lymphomas and can induce lymphoproliferative disease [195]. T cells over-expressing miRC-1 also have a propensity for increased activation, proliferation and effector cytokine production under non-polarizing, T_H_1 or T_H_2 conditions in vitro [194,195]. Furthermore, mice that lack miRC-1 in CD4^+^ T cells have reduced T_H_17 differentiation and effector function, and are protected from autoimmunity [196] and severe colitis [197]. In those papers, miR-19b-3p enhanced T_H_2 and T_H_17 differentiation by repressing *Pten*, a significant influencer of T cell biology [198], *Ikzf4,* and the pro-apoptotic protein *Bim* [194,195,197]. miRC-1 is also required for T_REG_ cells to prevent autoimmune disease [196]. Together these data indicate that miRC-1 miRNAs are broadly necessary for T cell function, irrespective of their differentiation program, primarily via the regulation of *Pten*.

It is worth noting that the miRC-1 gene is homologous to the miR-106a~363 cluster and the miR-106b~25 cluster, both situated in different chromosomes in mammals. Together, these three clusters can give rise to 13 distinct mature miRNAs, a genomic organization that is highly conserved in all vertebrates [199]. In spite of this, their possible relevance due to conservation, the organization of these miRNAs has made it challenging to determine the contribution of each one in the pre-CRISPR era of research, where genetic manipulation restricted the possibility of refined genetic mutations, and the majority of data on this important class of miRNAs derives from information on the entire cluster, rather than the individual mature miRNAs.

#### 3.3.9. miR-23~27~24 Cluster

The miRNA cluster miR-23~27~24 is another group of miRNAs that regulate T cell activation, differentiation and effector function, but it is not found in tissues affected by type-2 disease. These miRNA s are worth discussing as some investigators have discovered a strong link between them and the establishment of an adaptive type-2 immune response in murine models, as they target many of the essential nodes of Th2 cell differentiation and effector function, like *Gata3* and *il4* genes. Two locations in the mammalian genome encode for this family of miRNAs: one is the miRNA-cluster 11 (miRC-11) and one is the miRNA-cluster 22 (miRC-22). Collectively, these clusters produce five miRNAs: miR-23a-3p and miR-23b-3p, miR-27a-3p and miR-27b-3p, and miR-24-3p.

Although expressed ubiquitously, miRNAs of this clade are most abundant in lymphocytes [200]. As mentioned before, genomic engineering of miRNAs in tight proximity is often difficult with classic genomic tools previous to CRISPR. Therefore, the production of miRNA-deficient mice have so far only looked at the effect of depletion of the entire cluster. miR-27a/b-3p were initially discovered as a repressor of TCR–signaling and T cell activation [201]. In experiments where AGO proteins are cross-linked to their target mRNAs (AGO HITS-CLIP), putative miR-27a/b-3p targets were identified in T cell lines. Among the validated targets, transcription factors *Nrf2/Nfe2l2* and *Aff4*, as well as T cell effector molecules *Sema7a*, *Grb2*, and *Ifng* [201] were identified. The direct control of these factors by miR-27a/b-3p may explain how this miRNA functions as repressor of CD4^+^ T cell activation. In CD8^+^ T cells, miR-23a/b-3p (another miRNA of this cluster) targets *Lamp1*, a gene involved in CTL degranulation [202], yet again suppressing T cell function. In T_REG_ cells, the miR-23~27~24 cluster was found to be highly expressed [203], and inducible following TGFβ stimulation [202]. In the context of type-2 immunity, a more complex role emerges, as two studies found that the whole miR-23~27~24 cluster (rather than each individual miRNA) do not significantly regulate T helper cell activation, but rather modulate their differentiation. Deletion of these clusters did not affect T cells homeostasis, but caused an exacerbated type-2 immune response in two different lung allergy models following airway challenge [203,204]. In fact, in primary CD4^+^ T cells, miR-27a/b-3p directly represses *Gata3*, *Ikaros Family Zinc Finger 1* (*Ikzf1*) and *Nuclear Factor of Activated T-cells 2* (*Nfact2*), all factors necessary for IL-4 production and T_H_2 effector function. Similarly, and more directly, miR-24a/b-3p can target *Il4* mRNA. Importantly, these effects seem to be independent from TCR-mediated cellular proliferation or cell death, different from the original description [201]. Although the miR-23~27~24 cluster has not been found in tissues of type-2 disease, the literature has repeatedly pointed towards their powerful role in T cell activation and modulation and allergic asthma. miR-23~27~24 may therefore represent another therapeutic potential.

A visual summary of some of the discussed miRNAs—organized in tissue of origin and biological function—is presented in Figure 5 and Figure 6.

## 4. Conclusions and Future Perspectives

Type-2 immune-associated diseases, including asthma affect a growing percentage of the population with significant unmet medical needs. Although new therapeutic options are emerging, including Dupilumab (targeting IL-4α and disrupting IL-4 and IL-13 signaling) [205] and Tezepelumab (targeting TSLP) [206], new and alternative therapeutic targets, modalities and interventions are required to ensure we have an array of therapeutic options to match the most appropriate drug with patients. Targeting RNA species, including small and large mRNA [207] may represent one such new class of therapeutic agents. As we better understand the physiology, immunology and molecular biology of type-2 diseases, our therapeutic repertoire can, and should, equally expand. Endotype pathways, linking molecular mechanisms to a pathways, symptoms and clinically-relevant endpoints that provide benefit to patients are slowly emerging. Within the sphere of type-2 immune responses covered in this review, we can add lncRNA and miRNA species to these endotype pathways. Although much more work is required to identify, characterize and ascertain the function of lncRNAs in type-2 immunity and disease pathogenesis, the cell-type specificity of lncRNAs is a very attractive feature, allow us to dramatically increase the therapeutic index if a pathogenic lncRNA indeed has restricted expression. For example, if LincR-Ccr2-5′AS is indeed T_H_2 cell restricted and functionally relevant, then developing therapeutic modalities to target LincR-Ccr2-5′AS may have limited, if any untoward liabilities. Synthetic small-RNAs are currently being explored in clinical trials for a variety of indications, illustrating how miRNA delivery to patients is already a validated strategy. As outlined above several miRNAs have exciting potential as experimental candidates in type-2 diseases. For example, IL-13-mediated down-regulation of miR-449 appears to contribute to the pathogenic function of IL-13 in the airways leading to goblet cell hyperplasia and mucus hypersecretion. IL-13 is a validated target, and it is tempting to speculate that targeting downstream components, including miR-449, may be therapeutically viable options. Synthetic miR-449, which may prevent the differentiation of mucous-secreting cells [123], could be delivered locally, thanks to the development of nanoparticle-aided inhaled drug delivery, and may reduce mucus secretion without compromising other IL-13 mediated responses. Alternatively, upstream pathways that appear to be broadly required for immune cell development and activation, including mirR-155 and miR-21, could have more wide-reaching effects, but carry with them more liabilities. Local delivery may overcome some of these, but the full cost:benefit analysis of targeting miRNAs in general, and miR-155 and miR-21 in type-2 diseases specifically, may not be fully understood until they are clinically tested. 

## Figures and Tables

**Table 1:** **miRNAs differentially regulated in type-2 disease.** List of up-regulated (top) and downregulated (bottom) microRNAs found in human and mouse tissues in different type-2 diseases. Data are reported from independent investigators who looked at a panel of miRNAs in an unbiased fashion (via microarray, RT-qPCR screening or smallRNA-Seq) and compared healthy to inflamed tissue. The presence of the same miRNAs in both human disease and mice models, or in different type-2 disease, highlights its role as a ‘type-2-associated miRNA’.

		Human Type-2 Diseases				Mouse Type-2 Diseases			
		Skin		Respiratory Tract		Skin	Respiratory Tract		
miRNA	Function	Atopic Dermatitis	Psoriasis	EoE * (Esophagus Brushing)	Asthma (Airway Brushing)	Atopic Dermatitis	IL-13Tg ^#^ (Whole Lung)	Allergic Asthma ^%^ (Whole Lung)	
**miR-142**	immune-modulator	Sonkoly, E. Vennegaard, M.T.		Lu, T. X.		Vennegaard, M.T.	Lu, T.X.		**UP-REGULATED**
**miR-223**	anti-inflammatory	Sonkoly, E. Vennegaard, M.T.		Lu, T. X.		Vennegaard, M.T.			
**miR-21**	oncogene / pro-inflammatory	Vennegaard, M.T.	Joyce, C.E.	Lu, T.X.	Wu, X.-B	Vennegaard, M.T.	Lu, T.X.	Mattes, J.	
**miR-126**	pro-inflammatory / T cell suppr.				Wu, X.-B			Collison, A	
**miR-135**	unclear	Sonkoly, E.	Joyce, C.E.						
**miR-146**	anti-inflammatory	Vennegaard, M.T.		Lu, T.X.		Vennegaard, M.T.	Lu, T.X.		
**miR-92**	anti-apoptotic			Lu, T.X.	Solberg, O.D.				
**miR-1268**	unclear		Joyce, C.E.		Solberg, O.D.				
**miR-222**	oncogene / DC maturation		Joyce, C.E.	Lu, T.X.					
**miR-31**	unclear	Sonkoly, E.	Joyce, C.E.						
**miR-149**	anti-inflammatory				Solberg, O.D.				
**miR-33**	unclear		Joyce, C.E.						
**miR-155**	oncogene / modulator	Sonkoly, E.						Okoye, I	
**mR-149**	anti-inflammatory	Sonkoly, E. Vennegaard, M.T.		Lu, T.X.		Vennegaard, M.T.			
**miR-193**	anti-tumorigenic anti-inflammatory	Sonkoly, E. Vennegaard, M.T.				Vennegaard, M.T.			**DOWN-REGULATED**
**miR-30**	unclear	Sonkoly, E.		Lu, T.X.	Solberg, O.D.				
**miR-33**	unclear	Vennegaard, M.T.				Vennegaard, M.T.			
**miR-181**	anti-inflammatory	Vennegaard, M.T.				Vennegaard, M.T.			
**miR-34/449**	cilia differentiation				Solberg, O.D.			Yin, H.	
**miR-99**	anti-inflammatory	Sonkoly, E.			Solberg, O.D.				
**miR-195**	anti-inflammatory	Sonkoly, E.					Lu, T.X.		
**miR-26**	anti-inflammatory	Sonkoly, E.			Solberg, O.D.				
**miR-101**	pro-inflammatory	Sonkoly, E.					Lu, T.X.		
**miR-365**	anti-inflammatory	Sonkoly, E.		Lu, T.X.					

* EoE: Eosinophilic Esophagitis, ^#^ IL-13Tg: mouse model where IL-13 is over-expressed in tissue, ^%^ OVA and house dust mite models are included in this list.
